# Supplementary material for: Superelastic graphene aerogel-based metamaterials
Source: Nat Commun. 2022 Aug 5;13:4561. doi: 10.1038/s41467-022-32200-8 (PMC9355988; doi:10.1038/s41467-022-32200-8)
Supplement: Supplementary file 1 — Supplementary Information [file 41467_2022_32200_MOESM1_ESM.pdf]

Supplementary Information

Superelastic graphene aerogel-based metamaterials

Wu et al.

Supplementary Information

Contents

S1. Supplementary Notes

S1.1 Establishment of angle resolved radial distribution function.

S1.2 Molecular dynamics simulation.

S1.3 Finite element calculation

S2. Supplementary Figures

S2.1 Polymerization and imidization procedures of PI.

S2.2 Fabrication of PI nanofibers.

S2.3 Physical parameters of the PAA nanofibers and PI nanofibers.

S2.4 Chemical structures of GO and GmA.

S2.5 Structures of fiber-reinforced graphene walls.

S2.6 SEM image of GmA structure with long range orientation morphology and ordered lamellar structure.

S2.7 Compressive stress-strain curves of GmA and PGA at strain of 50%.

S2.8 Photographs of GmA under 90% compression and after release.

S2.9 Fatigue-resistance tests of GmA at different strain.

S2.10 Extreme compression of GmA.

S2.11 Tensile stress-strain curve of GmA.

S2.12 Photos of GmA under bending and folding deformation.

S2.13 Ashby plots for various types of materials.

S2.14 SEM observation of the compression processes of GmA and PGA at different scales.

S2.15 Models of RDF and ARRDF.

S2.16 SEM images of GmA and PGA with corresponding texture recognition ellipses under different compression states.

S2.17 Schematic of orientation angles of ellipse.

S2.18 ARRDF analysis.

S2.19 The values of ARRDF at different pixel distance.

S2.20 GmA with different thickness of graphene walls.

S2.21 The stress-strain curves of GmA with different fiber content at strain of 50%.

S2.22 Stress-strain curves of GA with different density of fibers

S2.23 Configurated GmAs with Negative Poisson’s ratio during uniaxial compression.

S2.24 Configurated GmAs with Positive Poisson’s ratio during uniaxial compression.

S2.25 The simulation results of the Poisson’s ratio as a function with the compression strain according to the finite element calculation.

S2.26 Normalized resistance ΔR/R0 values at a compression strain of 20%.

S2.27 Magnetically actuated GmA.

S2.28 Thermal barrier performances of GmA.

S2.29 SEM images of GmA after 5 min open-flame test.

S2.30 Ceramic aerogels.

S2.31 SEM images of GO sheets with an average lateral size of 8-12 μm.

S2.32 Chemical characterizations of GO.

S2.33 Frozen mold consisting of polymethyl methacrylate (PMMA) plates and PDMS layer.

S2.34 Cross-section SEM images of GmA and PGA.

S2.35 TGA-MS spectrum.

S2.36 SEM images of GmA surfaces after laser-cutting and tearing.

S2.37 Structure characterizations of Fe_3O_4 nanoparticles.

S3. Supplementary Tables

S3.1 Comparisons of the compressive performances of carbon-based aerogels.

S3.2 GmA with transverse deformation (ε_{22}) variation under different longitude applied strain (ε_{11}).

S3.3 Comparisons of the Poisson’s ratio of GmA with other carbon-based aerogels.

S4. Supplementary References

S1. Supplementary Note

Supplementary Note 1

Establishment of angle resolved radial distribution function

Such an interesting bulk deformation process is observed in Fig. 4 and Supplementary Fig. 14. In order to confirm whether 1D reinforced 2D structure causes transformation of bending mode, we established a novel angle resolved radial distribution function (ARRDF) to capture the texture changes under different compression state from the SEM observation (Supplementary Fig. 15 & 16). Specifically, the ARRDF is inspired by the radial distribution function (RDF) in statistical mechanics, which could be wrote as *g(r)* to describe how density varies as a function of distance from a reference particle. Here, we defined each piece of texture is recognized by an ellipse with its orientation and length of long and short axis being recorded, and the minimum area for texture recognition is set to 100 pixels^2 if not specified. The angles with vertical axis of image are used to represent the orientation of ellipse. Taking the vertical axis as the reference orientation of ellipse, the orientation of ellipse can be split into 18 regions, i.e. 175 to 5, 5 to 15, 15 to 25, …, and 165 to 175 degrees (Supplementary Fig. 17). Within each angle region, RDF could be measured, which is therefore called ARRDF in this work and written as $g(r, \theta)$.

$$g(r,\theta)=\frac{1}{\rho}\left(\sum_{i\neq 0,\ |\theta_i-\theta|\le 5^\circ}\delta(r-r_i)\right) \qquad (1)$$

where $g(r, \theta)$ is the ARRDF, ρ is the density, r is radius. Ellipse 0 is fixed as the origin of the coordinates, which is excluded from the nearest neighbor analysis.

Accordingly, tracing the distribution of ellipse angles could calculate out the distorted tendency of the graphene walls, further revealing the deformation process in statistics. The corresponding texture recognition images are shown in Fig. 4j-m and Supplementary Fig. 16. As $g(r,\theta)$ are nearly symmetric with respect to 90 degrees, $g(r,\theta)$ at 10, 30, 50, 70, 90, 110, 130, 150 and 170 degrees under different compression states are shown in Supplementary Fig. 18. Comparing the ARRDFs of GmA and PGA, a general characteristic is that during the compression the ARRDFs continuously reduce along with pixel distance increasing, however, the decrease levels are quite different. To quantify, the values of ARRDFs at different pixel distance (500, 1000, 1500 pixel distance, Fig. 4n, o and Supplementary Fig. 19) are used for comparison, in which the values of ARRDFs at 90 degrees under different compressions are chosen to normalize results, i.e. the horizontal spatial correlation serves as a reference here, because whether being nanofiber reinforced or not could hardly change this correlation. In this way, the influence from image contrast and resolution could be excluded. As mentioned above, the minimum area for texture recognition is set to 100

pixels^2, however, to rule out the influence from texture recognition number, the minimum area for texture recognition in the sample without nanofibers reinforcement under 80% compression is set to 49 pixels^2 to make it have the same texture recognition number as the 50% nanofibers reinforced sample under 80% compression. From the comparison, it is obvious that under same compression condition, away from 90 degrees ARRDFs in nanofiber reinforced cases are larger suggesting the graphene sheets are relatively straighter, demonstrating an extreme buckling deformation to bulk deformation transformation taking place with nanofiber reinforcement.

Demonstration of bending transformation not only provides the evidence to validate our modeling, but also it uncovers the underlying mechanism of the better recoverability with nanofiber reinforcement. According to experimental observation (Fig. 4 and Supplementary Fig. 14), this is attributed to less graphene sheets breaking under large compression, most of which occurs in large bended regions. Since the bending to folding transformation could reduce the extremely bended regions statistically, it thus benefits recoverability.

Supplementary Note 2

Molecular dynamics simulation

LAMMPS is used to perform molecular dynamics simulations.[1] The coarse grain model proposed by Cranford and et. is used in this work,[2] and the interaction parameters are obtained in the same way, except the size of coarse grain is much larger. In our simulation, graphene sheet is set to 4 um size square according to experiments, and the coarse grain size is 0.25 um, which means one graphene sheet is composed by 17×17 coarse grain particles. The thickness of graphene sheet layer is set to 140 nm, and average 2.5 layers at same place, which thus recovers 350 nm skeleton thickness observed in experiments. Periodic boundary condition is used in our calculation model (Please see Fig. 5a), and its box size is 30 um ×10 um ×20 um. With all this geometry, the mass and interaction parameters for coarse grain particles could be obtained. To be specific, mass equals 0.000475 pg, harmonic potential for bonds and angles are used, and the spring coefficient for bonds k_T equals 33607020 pg us^{-2} (the equilibrium distance r_0 = 0.25 μm), the spring coefficient for in-plane angles k_{phi} equals 11725 pg μm^2 · μs^{-2} (the equilibrium angle φ_0 = 90°), and the spring coefficient for out-plane angles k_θ equals 15737 pg μm^2 μs^{-2} (the equilibrium angle θ_0 = 180°). When considering the effective thickness, if strain energy under tension is assumed to keep unchanged only k_θ changes N^2 times (N is the ratio between effective thickness and original thickness), since it is the only parameter related to the bending modulus, and $k_\theta \propto \frac{E}{N} \cdot (N \cdot t)^3 = E \cdot N^2 \cdot t^3$. Other interaction parameters $k_T = \frac{E}{N} \cdot (N \cdot t) = E \cdot t$, $k_{phi} \propto \frac{G}{N} \cdot (N \cdot t) = G \cdot t$, thus they could not change.

The harmonic potential is used to describe bonds and angles between coarse grain particles within one fiber. The bond spring coefficient $k_{T,\text{fiber}}$ = 78600 pg us^{-2} and equilibrium distance $r_{0,fiber} = 1$ μm, and angle spring coefficient $k_{T,\text{fiber}}$ =49 pg us^{-2} um^2 and equilibrium angle $\varphi_{0,fiber} = 180°$. Assuming single fiber mechanical property shares same Young's modulus of graphene sheets, all these parameters could be derived from experiments. To be specific, the around 200 nm typical diameter of fibers, the graphene sheet surface occupying ratio, and the density difference induced by including fibers are all taken into account. Note that one fiber represents multiple fibers in reality, since only in this way the number of particles in our simulation could be reduced to a computable range. The mass of fibers is estimated and taken 0.0088825 pg in our simulations.

Lenard Jones potential is used to depict Van der Waals interactions. Same surface energy (260 mJ m^{-2}) are used to both graphene sheets and fibers,[2] with different surface area the coarse grain particles of graphene sheets and fibers have different epsilon, i.e.

epsilon = 32.5 pg μm^2 μs^{-2} and epsilon= 8 pg μm^2 μs^{-2}, respectively. Both equilibrium distance related parameter sigma = 0.2 um.

The configuration of our calculation model is generated by ourselves and followed by a minimization and then a molecule dynamics simulation with NVT ensemble to relax the system, the obtained structure is shown in Fig. 5a. To simulate the compression process, every time shrinking the box in Z dimension is followed by a minimization. This is because the low density of this system, a direct molecule dynamics run could have particles losing problem, which leads to breakdown of the simulation.

Here, we normalized relationship of stress and strain to confirm the contribution of toughening derived from bulk deformation process. As shown in Fig. 5b, we firstly established a model of stress-strain curve (blue dotted curve), which describes an ideal compression process only with buckling deformation. As displayed in Fig. 5c, bulk deformation model is better for avoiding the extreme bending, which contributes to maintain the complete structure of graphene walls during long-term compression cycling, demonstrating a good fatigue-resistance property.

Supplementary Note 3

Finite element calculation

For finite element calculation, Abaqus is used to demonstrate the designability of Poisson's ratio based on this material, where same sized structure and stress-strain curve obtained in experiments are used. Note that experimental tensile and compression curve of graphene foam is directly taken as a numerical constitutive relationship, which corresponds to the low density foam constitutive model implemented in Abaqus. There two things need to be clarified. First, the relaxation effect included in this model is set to zero due to the slow loading rate in experiments. Second, this model assumes zero Poisson's ratio, which is reasonable according to observations.

Specifically, with large compression over 50%, mechanical instability leads to structure distortion, which is not observed in experiments. Thus, here only the variation of Poisson's ratio before 50% compression is shown. And both 9-hole and 3-hole samples with different hole sizes are shown, which gives positive and negative Poisson's ratio, respectively. In Fig. 6e and Supplementary Fig.23-25 the color is used to reflect the distribution of displacement magnitude. The displacement of the red dot along horizontal direction is used to calculated the Poisson's ratio. It should be noted that for samples with different hole size, the tracing point is the one which deviates its original position most, and as a result it might not always at the middle of the edge.

S2. Supplementary Figures

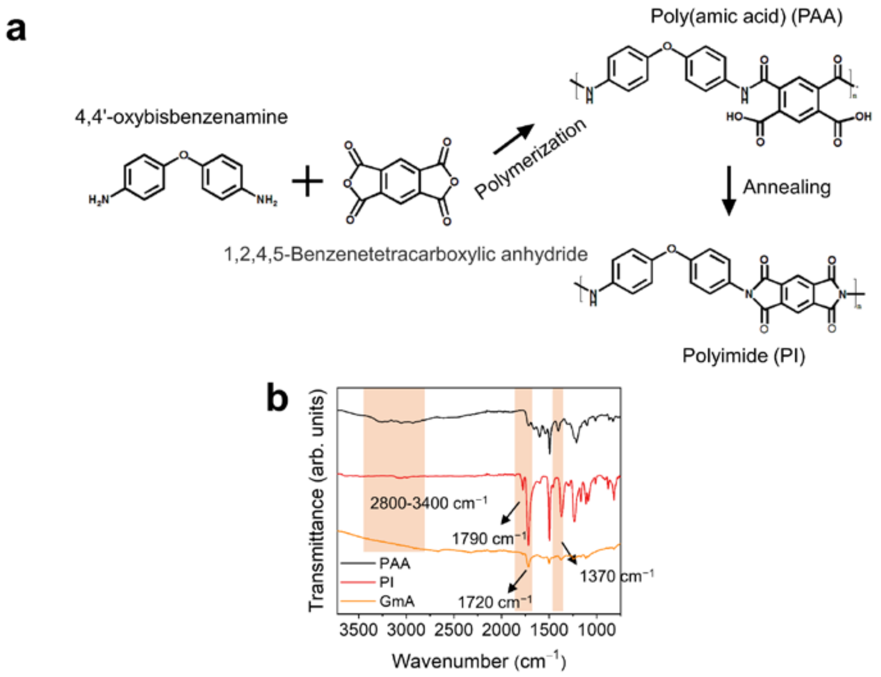

Supplementary Figure 1. Polymerization and imidization procedures of PI. **a** Chemical scheme for synthesis of PI. **b** Attenuated total reflection Fourier transform infrared (ATR-FTIR) spectra of PAA, PI, and GmA.

The PI nanofibers were fabricated by a two-step method. Firstly, the viscous PAA solution was polymerized by the dianhydride (PMDA) and diamine (ODA) in N,N-dimethylacetamide (DMAC). Subsequently, the PAA solution were electrospun into the nanofibers with a diameter of 243 ± 71 nm. Followed by the thermal imidization, the PAA nanofibers were converted into the PI nanofibers with a diameter of 207 ± 72 nm. The ATR-FTIR spectra of PAA, PI, and GmA were given in Supplementary Figure 1b, the typical peaks of the PAA included O−C=OH and NH_2 (3400−2800 cm^{-1}), C=O (1660 cm^{-1}), and C−NH stretch bands (1550 cm^{-1}). After the thermal treatment, some new identified peaks of the imidized PI appeared such as the asymmetric C=O stretching (1790 cm^{-1}), symmetric C=O stretching (1720 cm^{-1}), and C−N stretching (1370 cm^{-1}), indicative of the successful fabrication of the PI. Meanwhile, in the GmA, these characteristic peaks of PI could still be clearly observed, also demonstrating the PI nanofibers were well distributed in the graphene aerogels and maintained its original chemical structures.

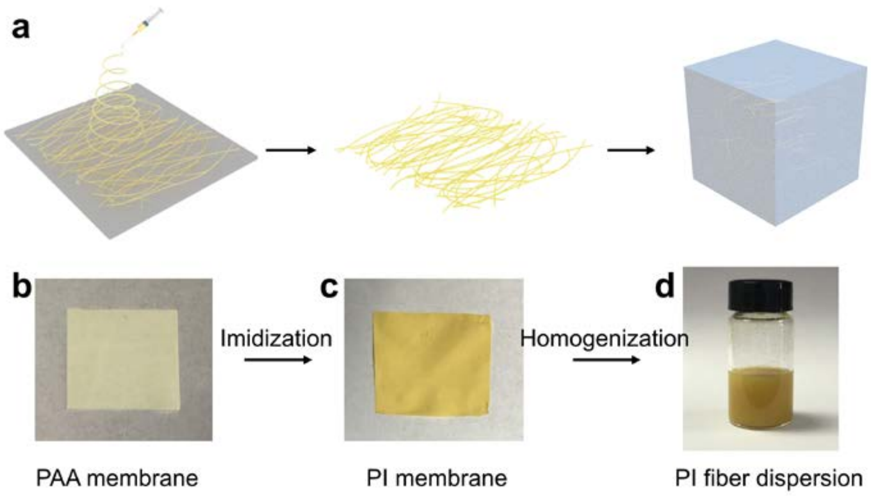

Supplementary Figure 2. Fabrication of PI nanofibers. **a** Schematic illustration of the fabrication of PI nanofiber dispersions. Photographs of **b** electrospun PAA membrane, **c** PI membrane, and **d** homogenized PI fibers dispersion stored in the glass bottle.

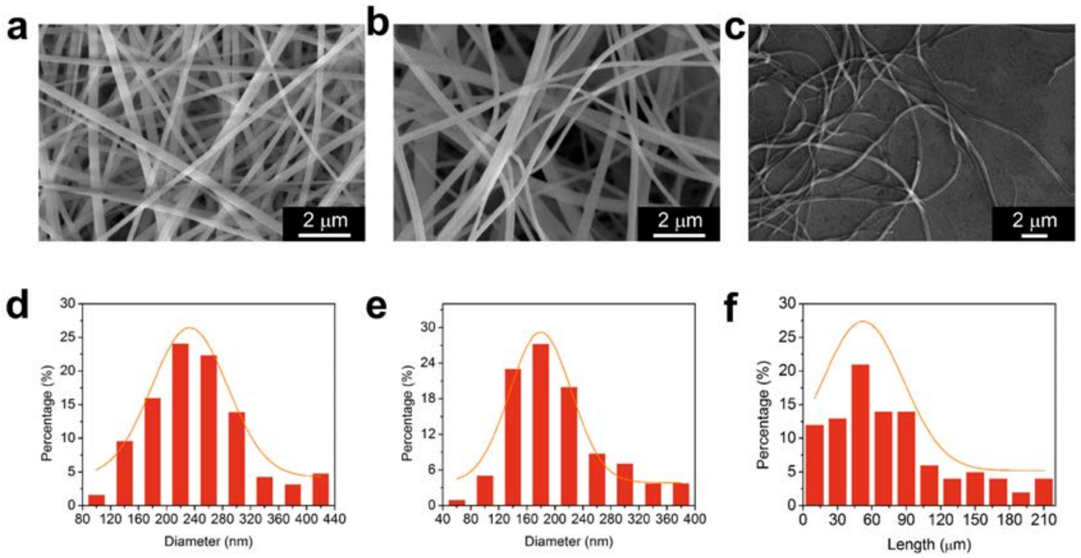

Supplementary Figure 3. Physical parameters of the PAA nanofibers and PI nanofibers. SEM images of **a** PAA nanofibers, **b** PI nanofibers, and **c** PI nanofibers after high-speed shearing. The histograms of **d** PAA nanofibers diameter distributions, **e** PI nanofibers diameter distributions, and **f** PI nanofibers length distributions.

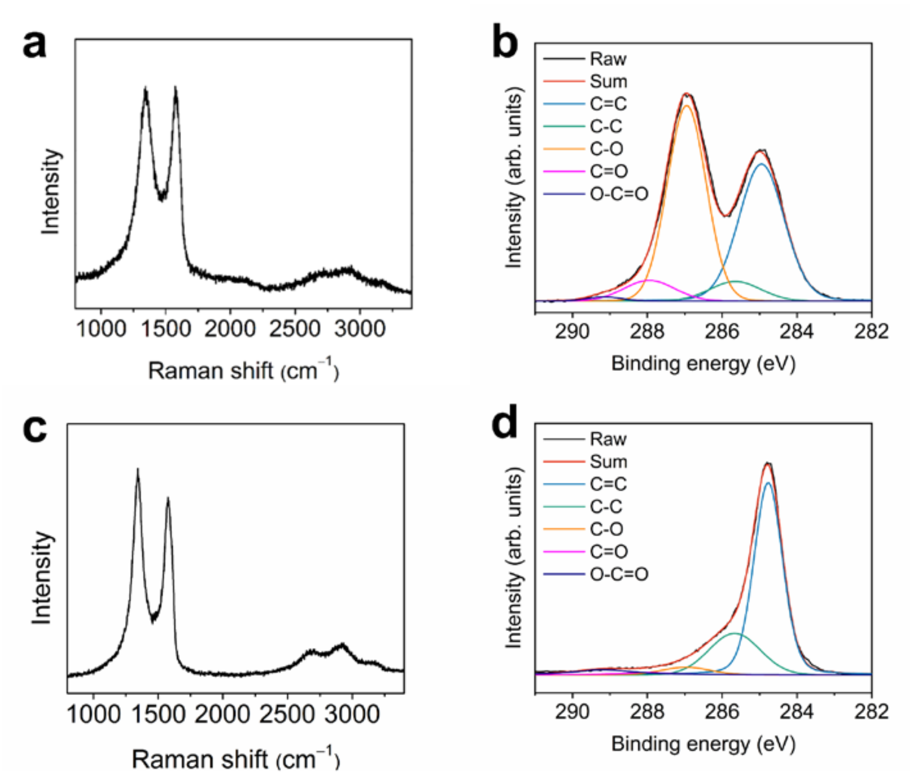

Supplementary Figure 4. Chemical structures of GO and GmA. **a, c** Raman and **b, d** C *1s* XPS spectra of GO and GmA.

The Raman spectra of carbon materials presents the typical D band (1330-1340 cm^{-1}) and G band (1580-1600 cm^{-1}). Their relative intensity ratio of I_D/I_G represents the average distance between defects (L_D) on carbon materials. The increasing L_D of GmA exhibits the removal of oxygenated groups and reduction of GO. Meanwhile, the greatly decreasing of the oxygen species on the XPS spectra also prove the well reduction of GO after annealing treatment.

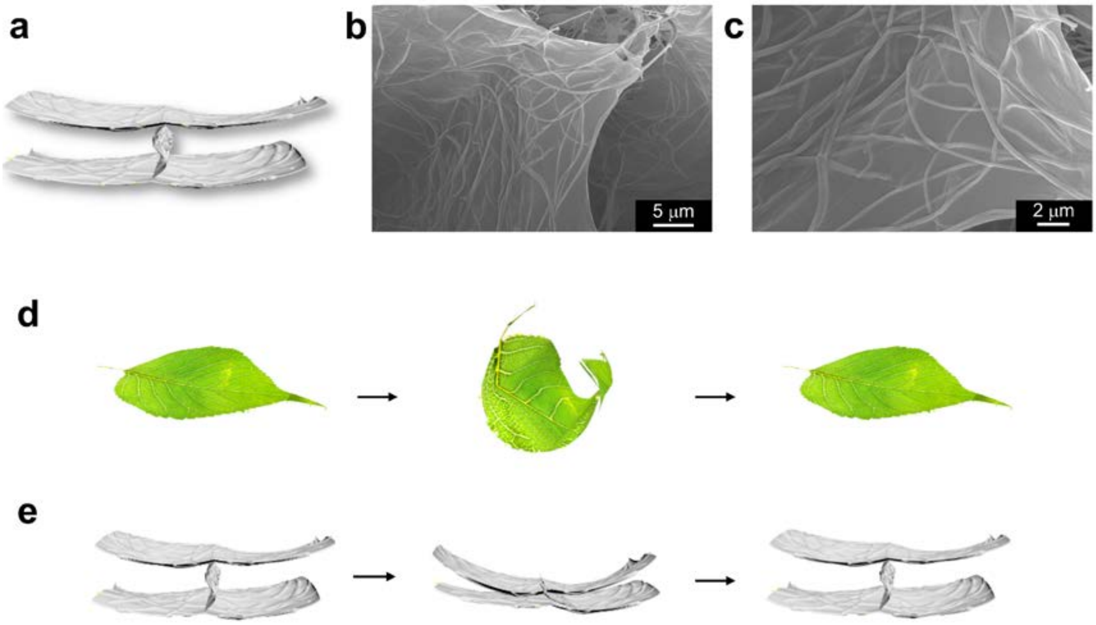

Supplementary Figure 5. Structures of fiber-reinforced graphene walls. **a** Schematic of fiber-reinforced graphene walls. **b, c** SEM image of fiber-reinforced graphene walls. **d** Schematic of the flexibility of a leaf. **e** Schematic of the flexibility of frameworks of fiber-reinforced graphene walls.

In nature, bio-composites composed by natural fibers is the most common strategy to reinforce their main structure at low cost. As shown in Fig. 2a, the leaf structure is a typical example consisting of veins and mesophyll. Here, 1D nanofiber reinforced 2D graphene sheet structure is similar with the nature strategy (Supplementary Fig. 5a-c), which utilizes graphene walls as the basic building blocks in the GmAs, and nanofibers as the reinforced skeletons to provide solid frameworks. Therefore, the GmAs can exhibit the good recovery capability like a leaf (Supplementary Fig. 5d,e).

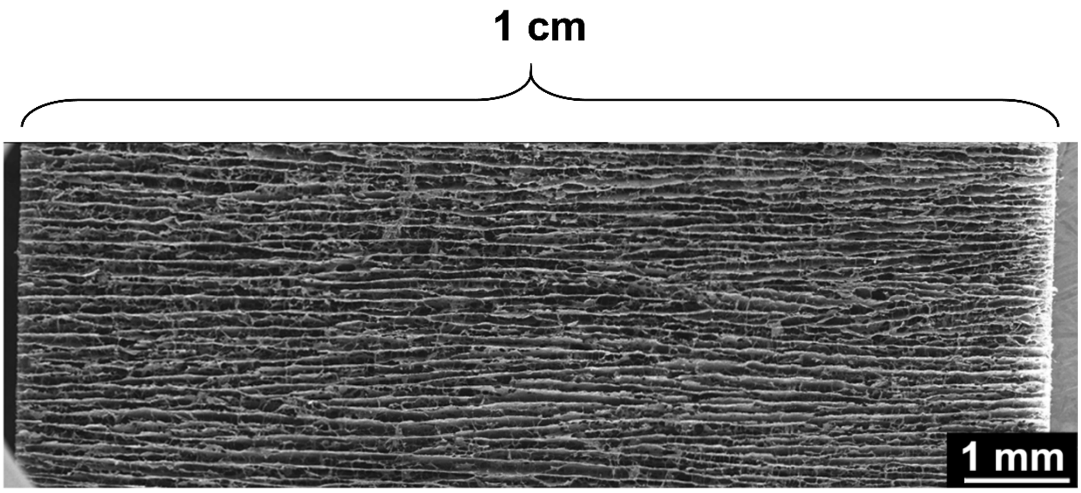

Supplementary Figure 6. SEM image of GmA structure with long range orientation morphology and ordered lamellar structure.

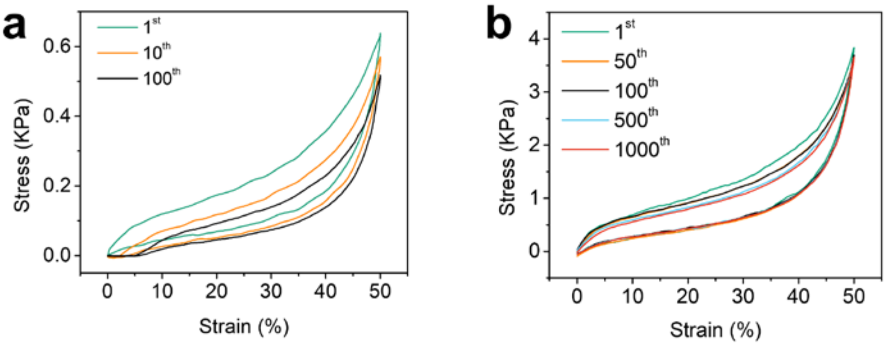

Supplementary Figure 7. Compressive stress-strain curves of GmA and PGA at strain of 50%. **a** 100 cyclic compression of PGA. **b** 1000 cyclic compression of GmA.

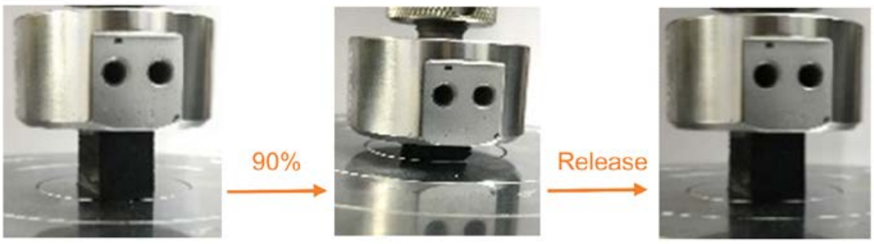

Supplementary Figure 8. Photographs of GmA under 90% compression and after release.

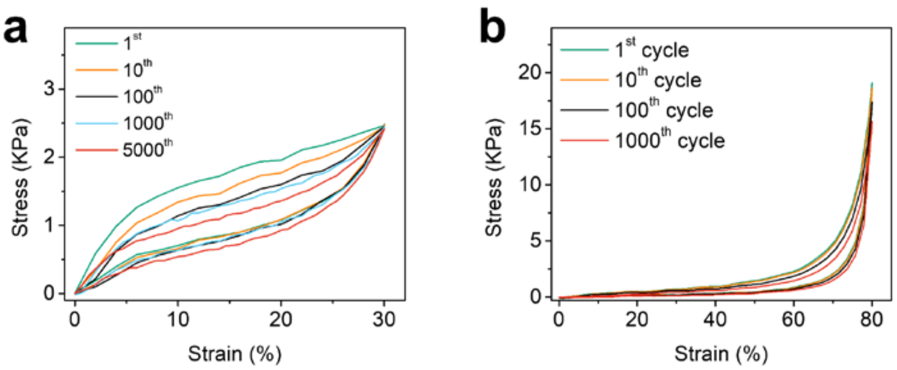

Supplementary Figure 9. Fatigue-resistance tests of GmA at different strain. **a** 5000 cyclic compression of GmA at 30% strain. **b** 1000 cyclic compression of GmA at 80% strain.

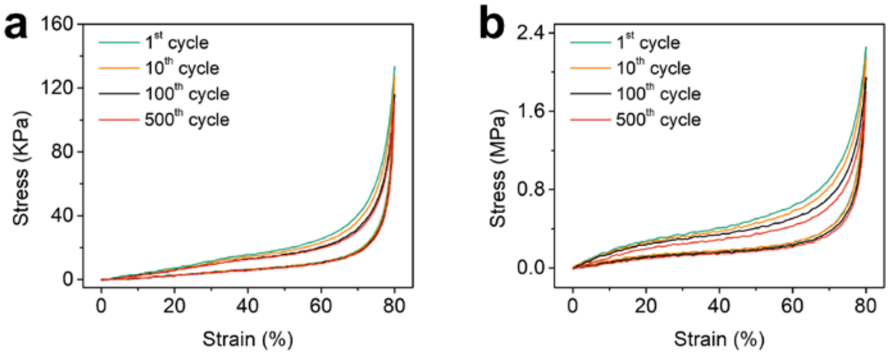

Supplementary Figure 10. Extreme compression of GmA. **a** 500 cyclic metal rod compression of GmA at 80% strain. **b** 500 cyclic blade compression of GmA at 80% strain.

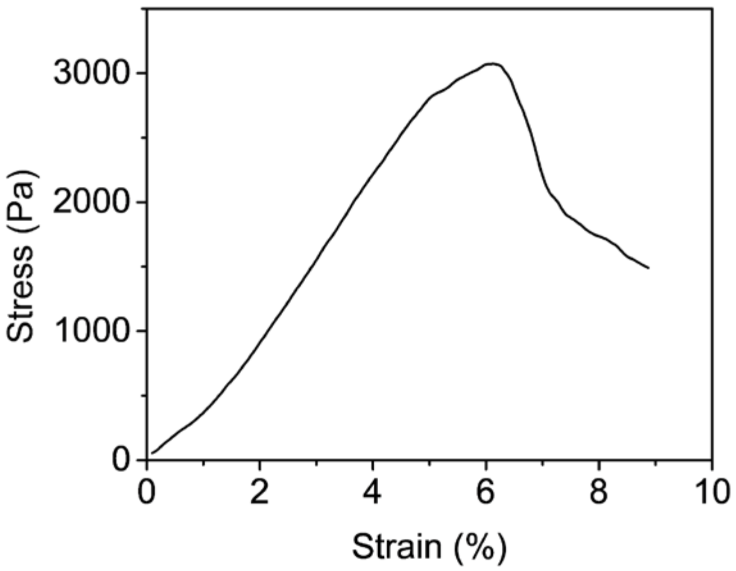

Supplementary Figure 11. Tensile stress-strain curve of GmA.

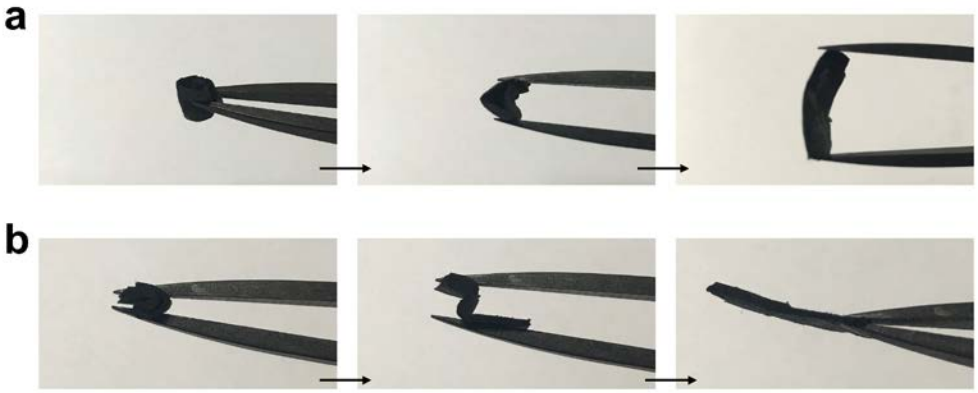

Supplementary Figure 12. Photos of GmA under bending and folding deformation. **a** The GmA under bending deformation and the recovery process. **b** the GmA under folding deformation and the recovery process.

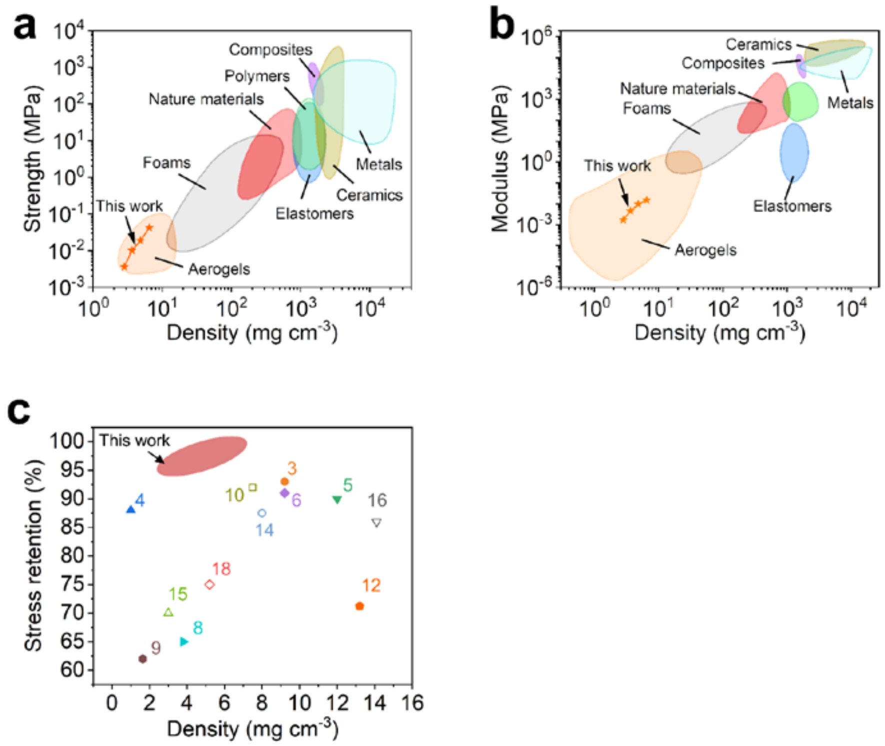

Supplementary Figure 13. Ashby plots for various types of materials. **a** Strength vs. density. The strength of GmA is the stress under 80% compression strain. **b** Modulus vs. density. The data was collected from references and CES EduPack 2019, ANSYS Granta © 2020 Granta Design. **c** Stress retention versus density of carbon aerogels under 50% compression strain.

As shown in the Ashby plots (Supplementary Fig. 13), aerogels are ultralight materials with a density typically less than 10 mg cm^{-3}. The strength and modulus of aerogels are mainly located in the lower left area of the figures. Among aerogels, GmAs are typical materials with good strength and modulus. Considering the excellent resilience (Supplementary Fig. 13c), the GmA is hopeful for further applications with good durability and processability.

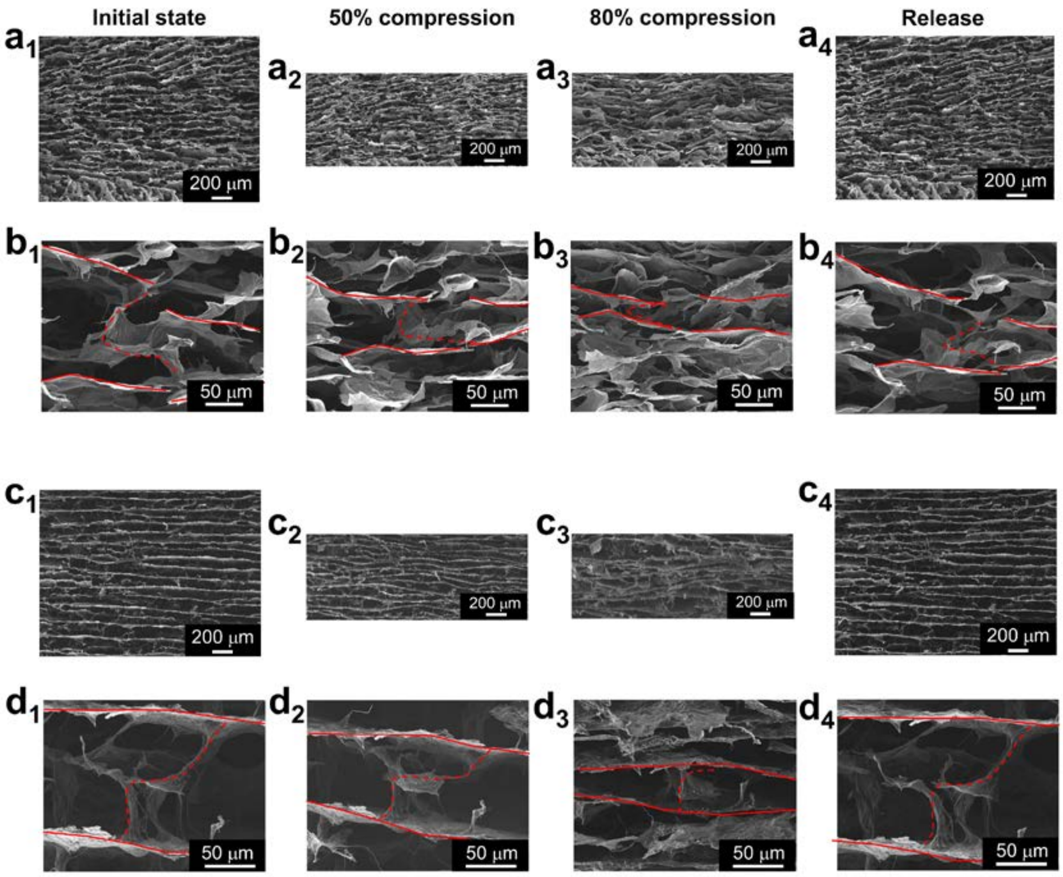

Supplementary Figure 14. SEM observation of the compression processes of GmA and PGA at different scales. **a, b** SEM images of compression process (initial state, 50% compression, 80% compression, release) of PGA. **c, d** SEM images of compression process (initial state, 50% compression, 80% compression, release) of GmA.

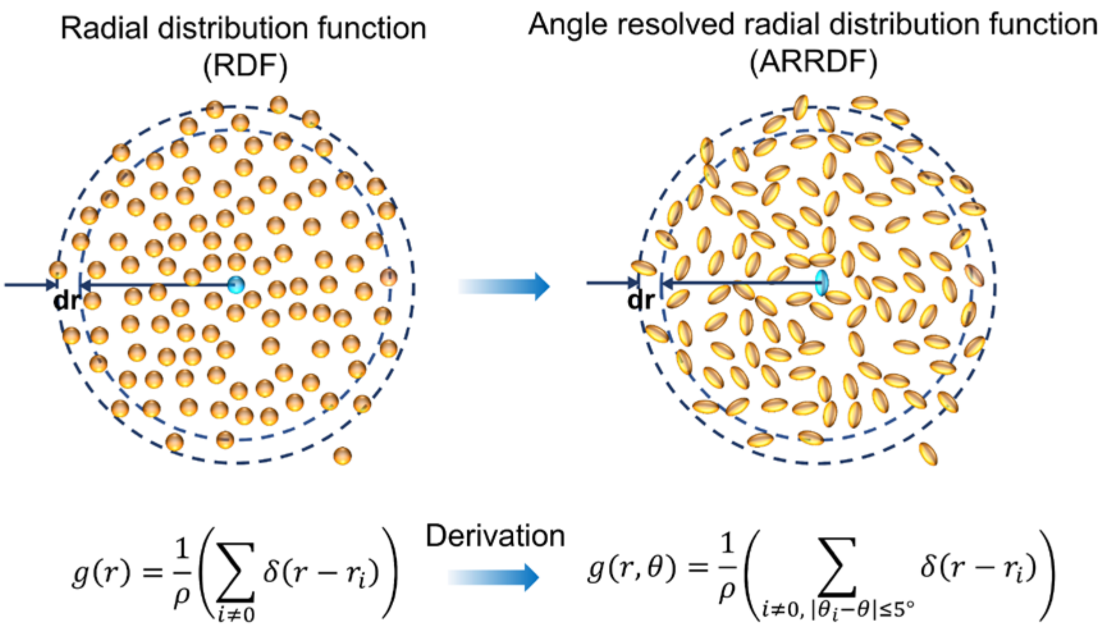

Supplementary Figure 15. Models of RDF and ARRDF.

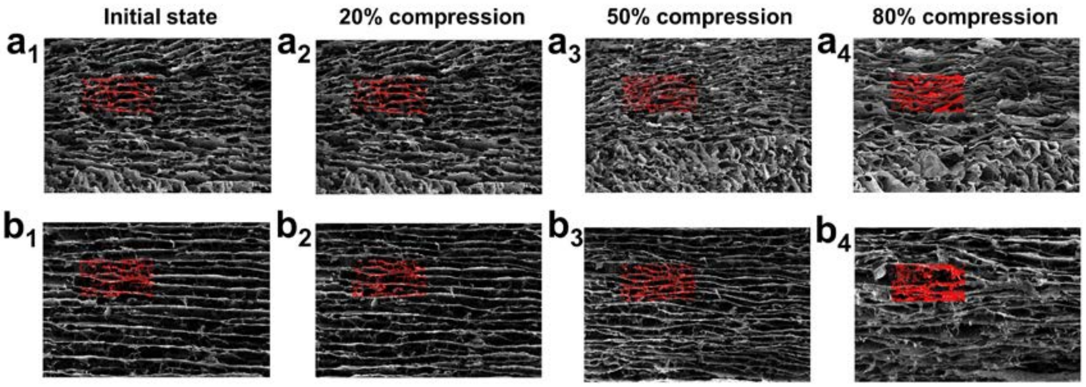

Supplementary Figure 16. SEM images of GmA and PGA with corresponding texture recognition ellipses under different compression states. **a** PGA. **b** GmA.

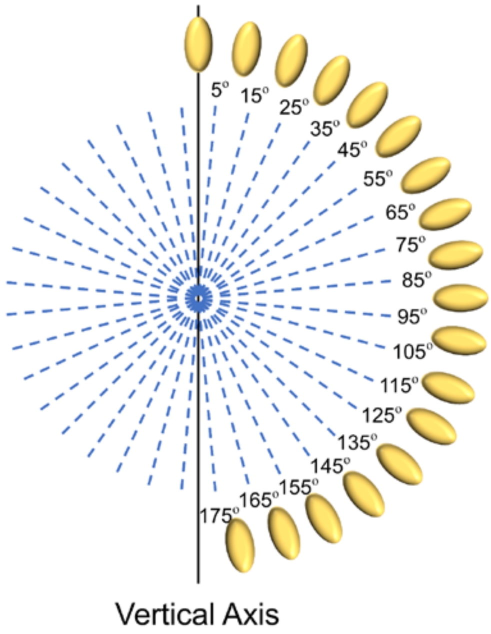

Supplementary Figure 17. Schematic of orientation angles of ellipse.

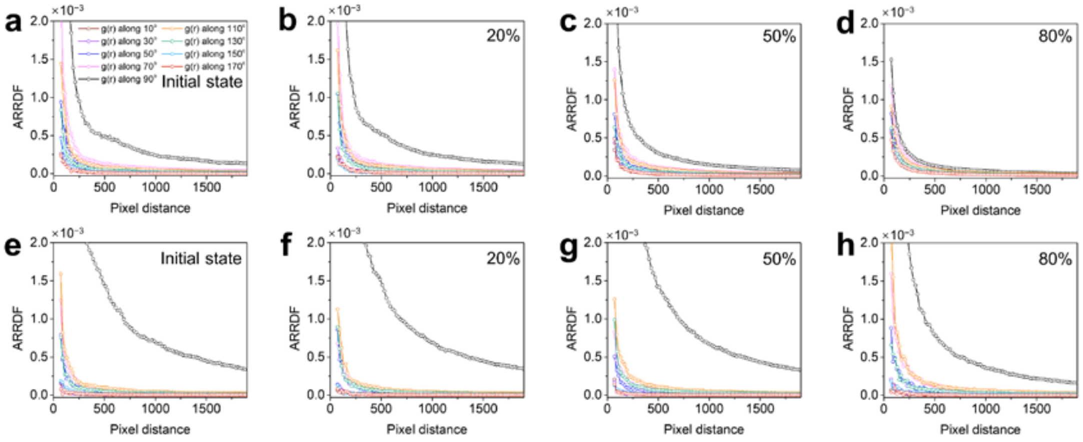

Supplementary Figure 18. ARRDF analysis. **a, b, c, d** ARRDF as a function of pixel distance of GmA under different compression state. **(a)** initial state. **(b)** 20% compression. **(c)** 50% compression. **(d)** 80% compression. **e, f, g, h** ARRDF as a function of pixel distance of PGA under different compression state. **(e)** initial state. **(f)** 20% compression. **(g)** 50% compression. **(h)** 80% compression.

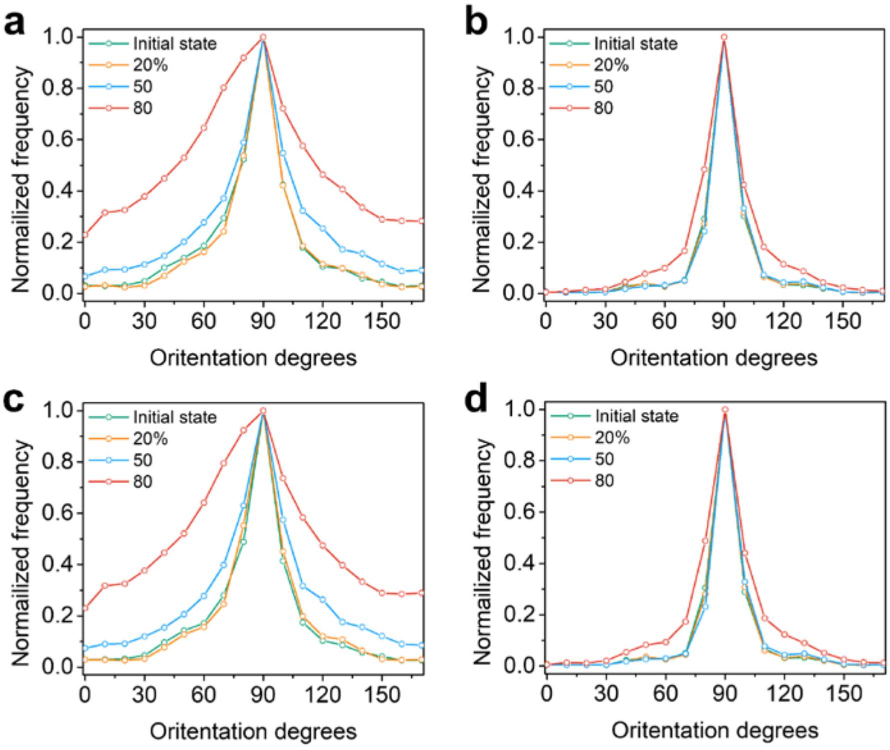

Supplementary Figure 19. The values of ARRDF at different pixel distance. **a, b** at 1000 pixel distance. **c, d** at 1500 pixel distance. **a, c** GmA. **b, d** PGA.

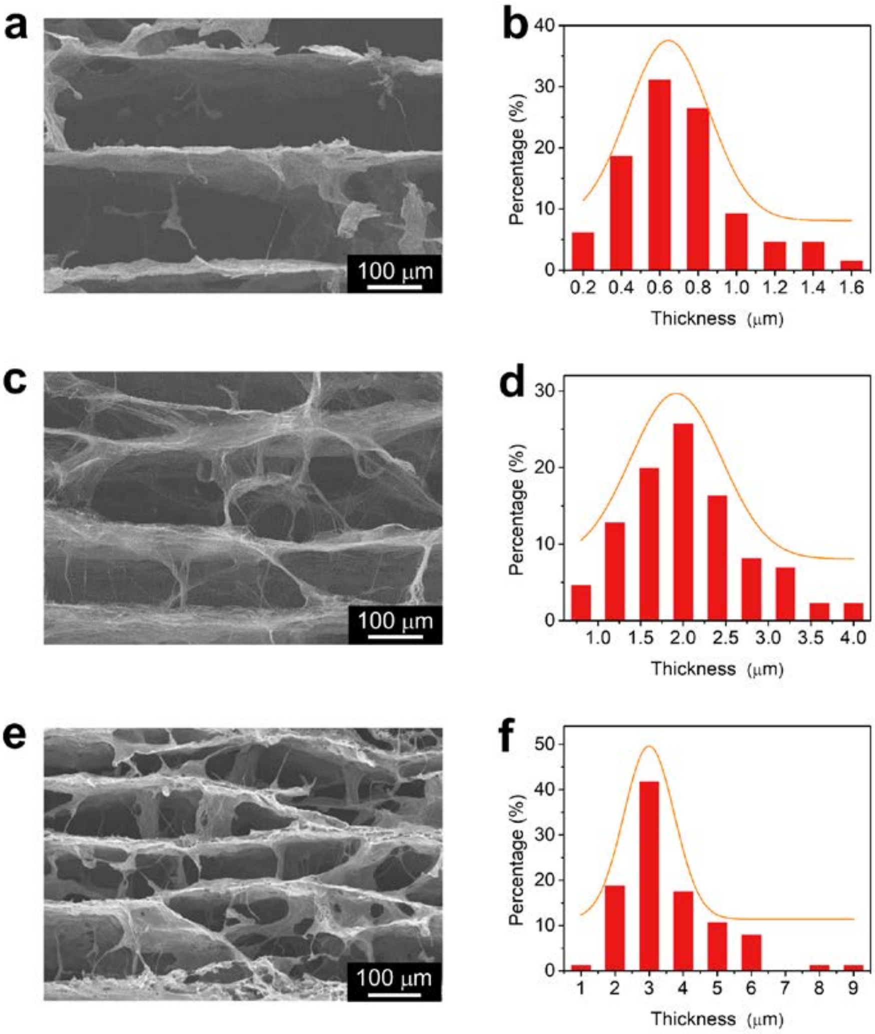

Supplementary Figure 20. GmA with different thickness of graphene walls. **a** SEM images of 0.1-GmA. **b** The histograms of thickness distribution of 0.1-GmA. **c** SEM images of 0.3-GmA. **d** The histograms of thickness distribution of 0.3-GmA. **e** SEM images of 0.5-GmA. **f** The histograms of thickness distribution of 0.5-GmA.

Here, in order to explore whether the fiber content would influence the mechanical performances of the GmA, we prepared three samples with different fiber contents, which rely on the feed weight ratio of PI fibers to GO. And the as-prepared GmAs were nominated as 0.1-GmA (weight ratio of PI fibers to GO is 1:10), 0.3-GmA (weight ratio of PI fibers to GO is 3:10), and 0.5-GmA (weight ratio of PI fibers to GO is 5:10), respectively.

Note that typical thickness means frequently observed thicknesses but not a mean value of thickness, since the graphene sheets are neither always nor strictly perpendicular to the lens of the microscope.

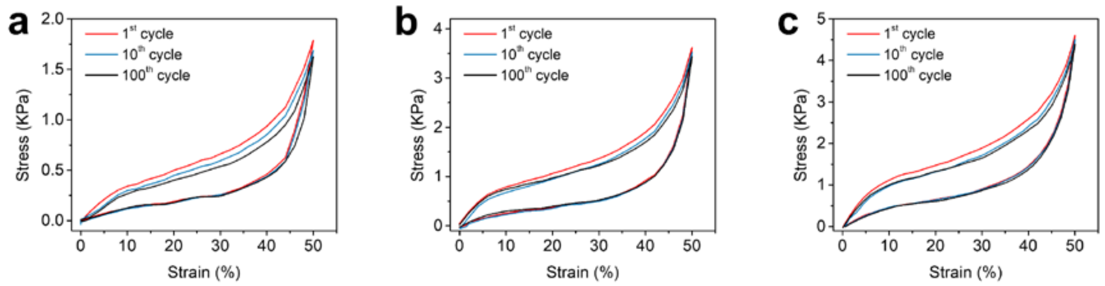

Supplementary Figure 21. The stress-strain curves of GmA with different fiber content at strain of 50%. **a** 0.1-GmA. **b** 0.3-GmA. **c** 0.5-GmA.

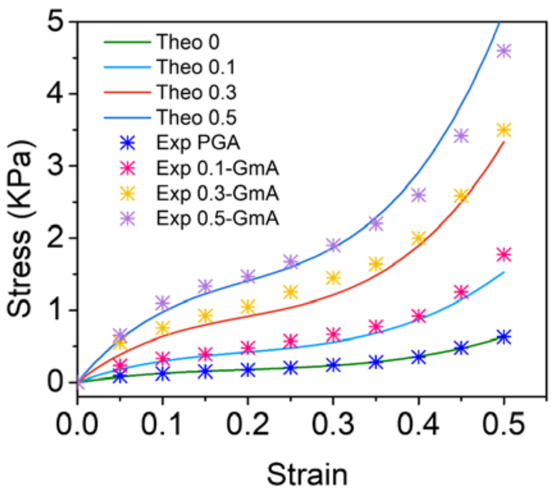

Supplementary Fig. 22. Stress-strain curves of GA with different density of fibers, where dots are experimental data with fiber density that takes 0, 0.1, 0.3 and 0.5, and solid lines are theoretical results (Theo 0, Theo 0.1, Theo 0.3, and Theo 0.5) according to Eq.(2).

The free energy density is given by

$$F_{int}(\varepsilon) = a\rho_f + b\rho_{2D}\rho_f + c\rho_f\rho_{2D}^2 + d\rho_f\rho_{2D}^3, \qquad (2)$$

where a= -289.83 kPa, b= 360.56 kPa, c= -199.33 kPa, and d= 41.68 kPa, nanofiber density ρ_f and ρ_{2D} graphene density under compression are represented by

$$\rho_f = (1+\varepsilon)\rho_{f;0},\ \rho_{2D} = (1+\varepsilon)\rho_{2D;0}\ ,$$

where and ε is compressive strain and $\rho_{f;0}$ and $\rho_{2D;0}$ are the corresponding densities of the nondeformed state. Nanofiber density $\rho_{f;0} = \rho_{fa;0} + 0.07$, where the added fiber density $\rho_{fa;0}$ takes 0, 0.1, 0.3 and 0.5 according to experiments, and 0.07 could be understood as the effective nanofiber density originating from crosslinking or entangling effects in the primitive graphene foam where no nanofibers are added. Note that density of graphene skeleton ρ_{2D} is taken as a density reference thus equals to one, for example $\rho_{fa} = 0.1$ means the added fiber density is 0.1 times of ρ_{2D}. As can be seen in Supplementary Fig. 22, experimental stress-strain curves of graphene foam with different density of nanofibers could be well described by our theoretical model (stress equals to the derivative of $F_{int}(\varepsilon)$ with respect to compressive strain ε).

Since the free energy density already include all relevant microscopic interaction ways, that of a typical microstructure i.e. local graphene skeleton, should have the same form but different parameters. Taking advantage of this form, Young's modulus E is found to be proportional to the initial value of fiber density $\rho_{f;0}$ (ρ_f at the undeformed state), which contribute to the enhancement of bending stiffness $D = Et^3/12(1-\nu^2)$, where t is the thickness of graphene skeleton and ν is Poisson's ratio.

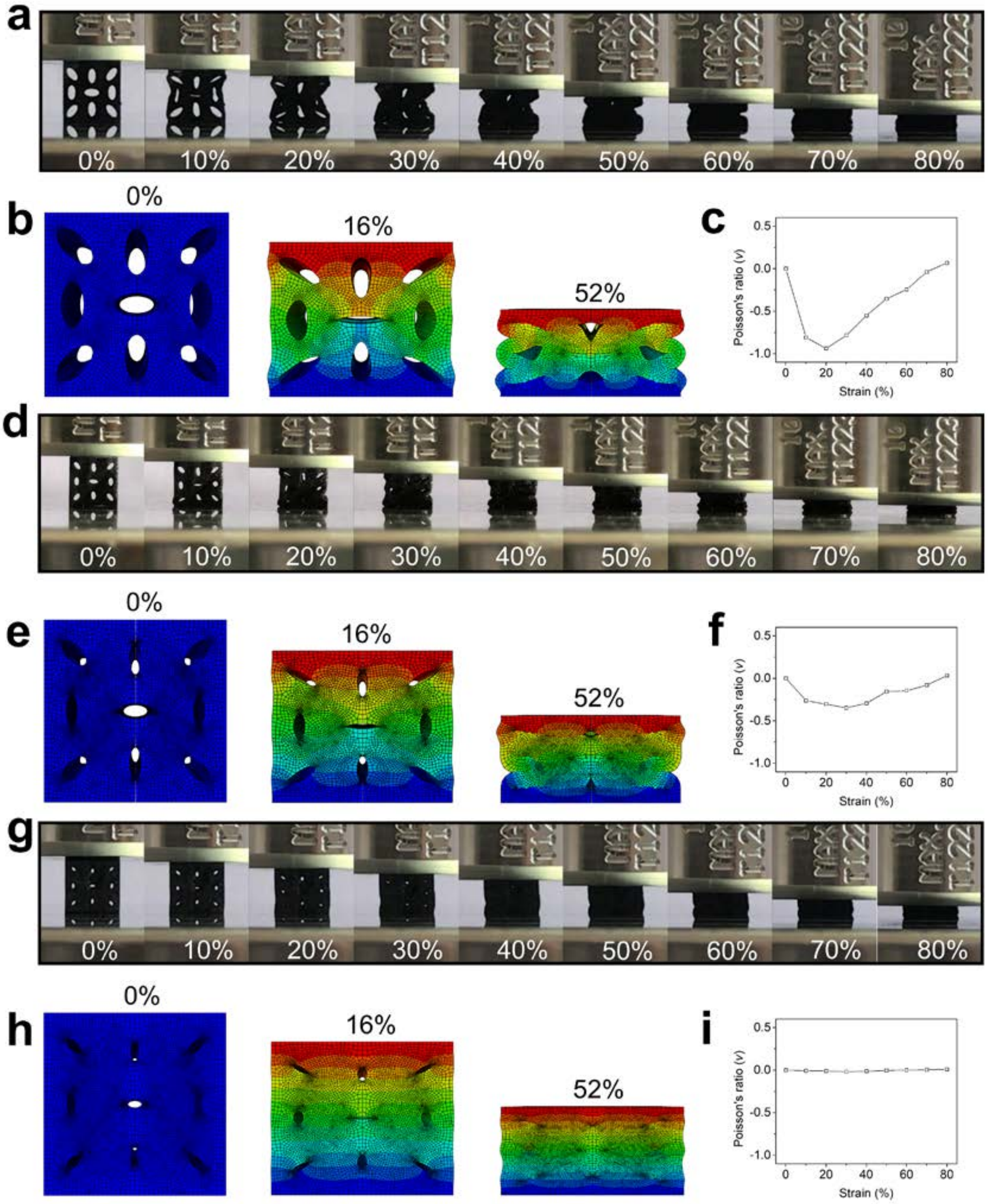

Supplementary Figure 23. Configurated GmAs with Negative Poisson's ratio during uniaxial compression. **a, d, g** The snapshots of cross-section views of GmA under compression state. **(a)** GmA$_{-1}$. **(d)** GmA$_{-0.7}$. **(g)** GmA$_{-0.4}$. **b, e, h** The finite element simulation process. **(b)** GmA$_{-1}$. **(e)** GmA$_{-0.7}$. **(h)** GmA$_{-0.4}$. **c, f, i** The Poisson's ratio as a function with compression strain. **(c)** GmA$_{-1}$. **(f)** GmA$_{-0.7}$. **(i)** GmA$_{-0.4}$.

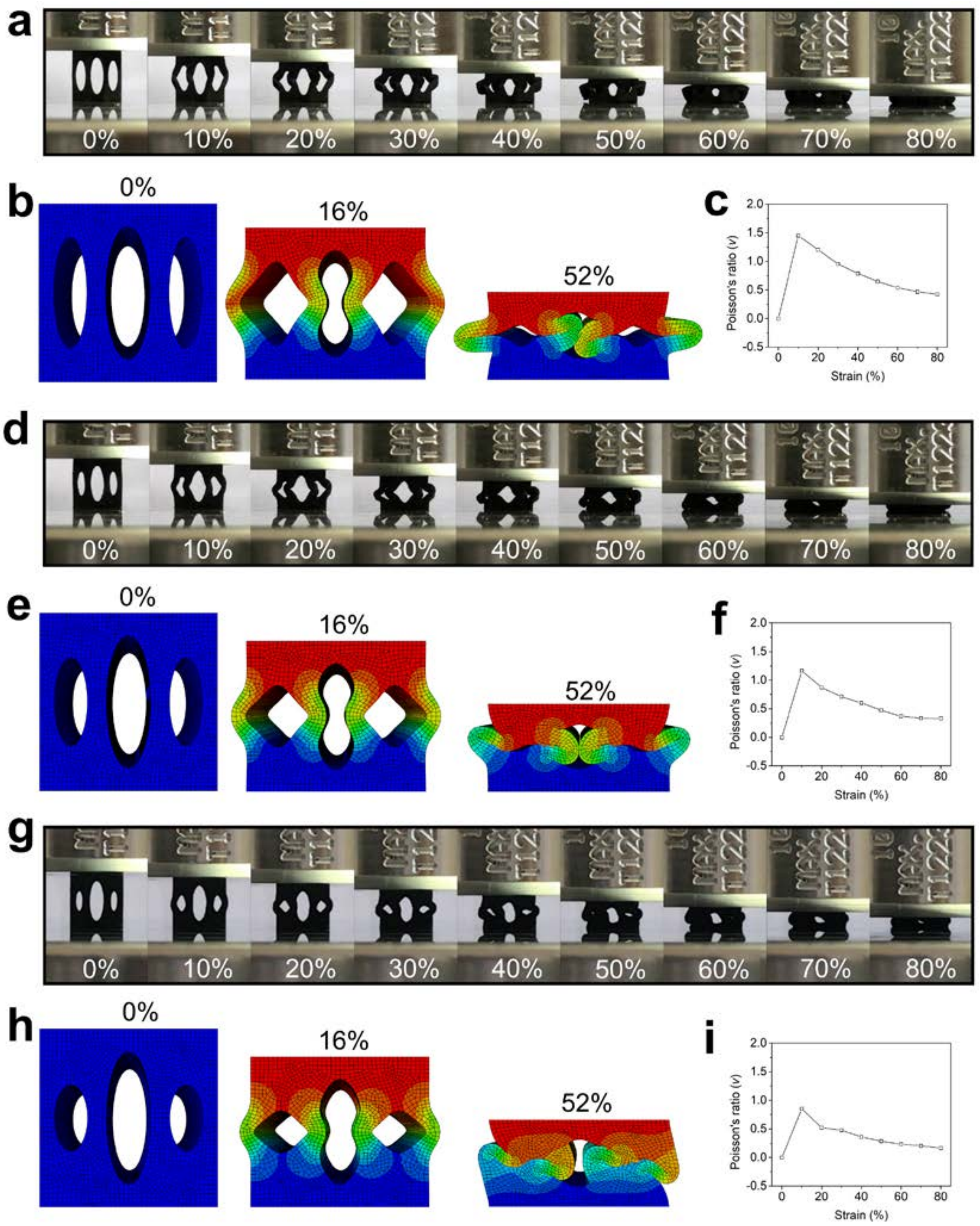

Supplementary Figure 24. Configurated GmAs with Positive Poisson's ratio during uniaxial compression. **a, d, g** The snapshots of cross-section views of GmA under compression state. **(a)** GmA_{+5}. **(d)** GmA_{+4}. **(g)** GmA_{+3}. **b, e, h** The finite element simulation process. **(b)** GmA_{+5}. **(e)** GmA_{+4}. **(h)** GmA_{+3}. **c, f, i** The Poisson's ratio as a function with compression strain. **(c)** GmA_{+5}. **(f)** GmA_{+4}. **(i)** GmA_{+3}.

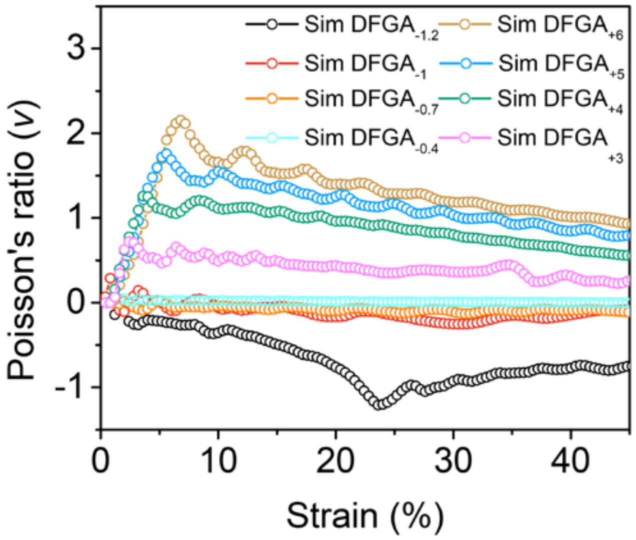

Supplementary Figure 25. The simulation results of the Poisson's ratio as a function with the compression strain according to the finite element calculation.

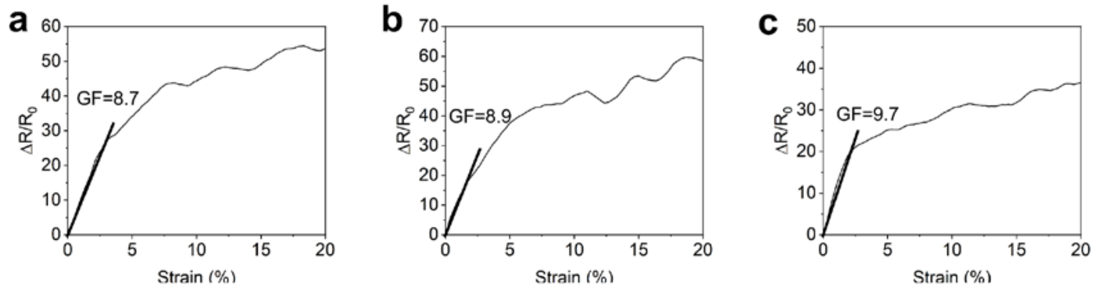

Supplementary Figure 26. Normalized resistance $\Delta R/R_0$ values at a compression strain of 20%. **a** GmA without holes. **b** GmA$_{-0.7}$. **c** GmA$_{-1.2}$. ΔR is the changing value of the resistance during compression. R_0 is original resistance without compression.

Compared with common materials, negative Poisson's ratio materials have superior shear resistance, indentation resistance, and fracture toughness, which makes them suitable for energy absorption applications, such as aerospace, defense, and sports protection. for the GmA with low density, although it can no longer guarantee its application in mechanical energy absorption, light skeletons will be more sensitive to stress and strain. Supplementary Fig. 26 shows under the condition of small strain, the GmA with negative Poisson's ratio, such as GmA$_{-0.7}$ and GmA$_{-1.2}$, exhibits larger gauge factor (GF) compared with the GmA without any holes, demonstrating the better sensitivity. The GF is calculated by $(\Delta R/R_0)/\varepsilon$, where ΔR is the resistance change under compression and R_0 is the resistance before straining. Additionally, the ultra-light GmA can be used as a platform to incorporate other particles to achieve more functionalities. The magnetically responsive actuator was demonstrated by the low density GmA in Supplementary Fig. 27.

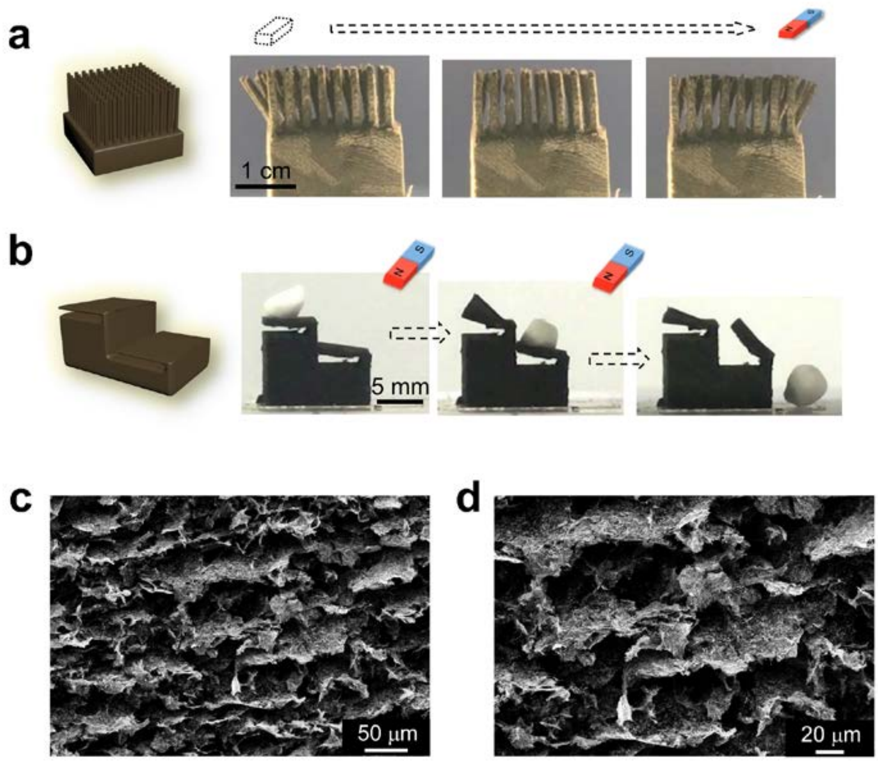

Supplementary Figure 27. Magnetically actuated GmA. **a** The column array responding with alternating magnetic field. **b** The stair springs down the small objects under the approaching magnet. **c, d** SEM images of GmA incorporated with Fe_3O_4 nanoparticles.

As demonstrated, the magnetically actuated GmA exhibits reversible deformation responsive to remote stimuli. The column array with the diameter of single column of 1 mm can exhibits a flexible wave-like deformation along with the movement of magnetic field. For the stair spring, the platform part of the aerogels can behave like a spring. Meanwhile, the fabricated framework retains the ordered structure with precise macroscopic morphology.

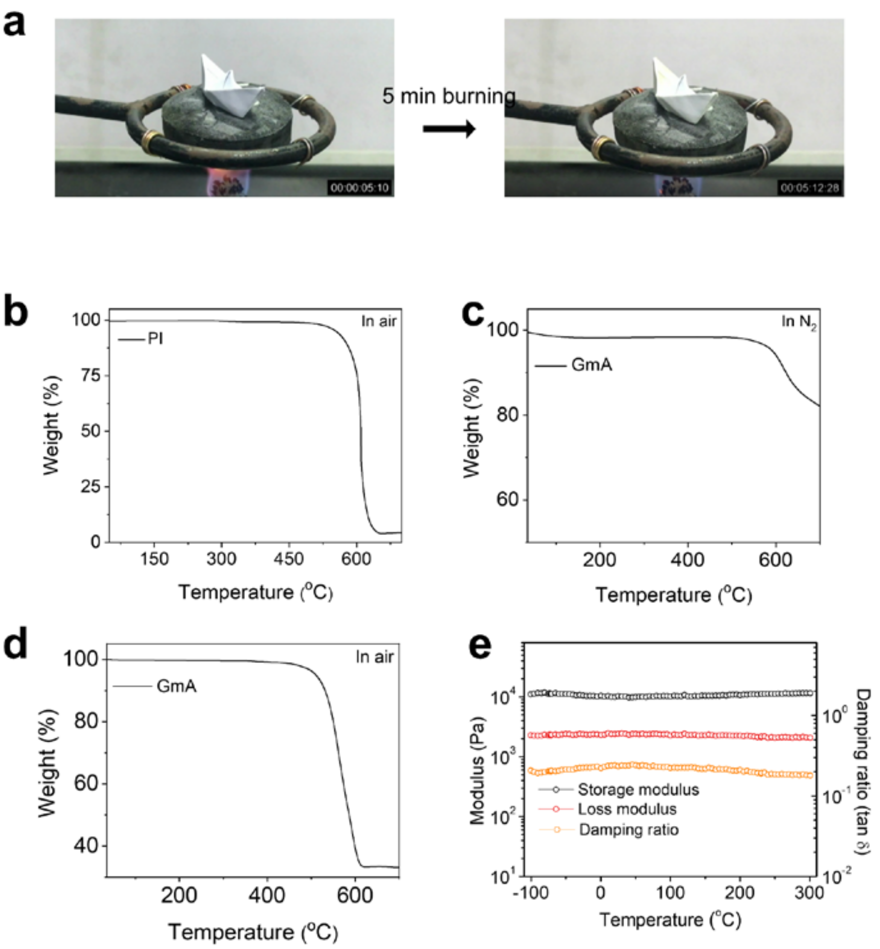

Supplementary Figure 28. Thermal barrier performances of GmA. **a** Paper boat against the flame burning of alcohol lamp for 5 min with a 1.3 cm thick GmA barrier. The thermogravimetric analysis (TGA) of PI and GmA. **b** PI fibers in air. **c** GmA in N_2. **d** GmA in air. **e** Temperature dependence of storage modulus, loss modulus, and damping ratio of GmA.

Supplementary Fig. 28a display the GmA can efficiently reduce the combustion rate in this open-flame test for 5 mins. The TGA measurements exhibit PI nanofibers are stable in air when the temperature is below 500 °C (Supplementary Fig. 28b). In a nitrogen atmosphere, the GmA are stable when the temperature is below 550 °C (Supplementary Fig. 28c). However, the TGA curve of GmA in air presents three distinct regions (Supplementary Fig. 28d). (1) In the process of increasing the temperature from 50 to 500 °C, the GmA has almost no weight loss (less than 1%), indicating that the reduced graphene oxide and PI nanofibers are stable below 500 °C. (2) As the temperature is higher than 500 °C, the weight loss of GmA begins again, which is derived from the oxidation reaction of reduced graphene oxide and decomposition of the polymer carbon backbone. (3) As the temperature is higher 600 °C,

the weight of residual carbon skeletons is constant.

The viscoelastic properties of the GmAs are characterized by the dynamical mechanical analysis (DMA). The nearly constant storage modulus, loss modulus, and damping ratio within a wide temperature range from −100 to 300 °C (Supplementary Fig. 28e) demonstrates that the elastic behavior of GmA is invariant with the temperature, which is an enthalpic elasticity rather than the entropic elasticity. Therefore, the GmA possess excellent temperature stability.

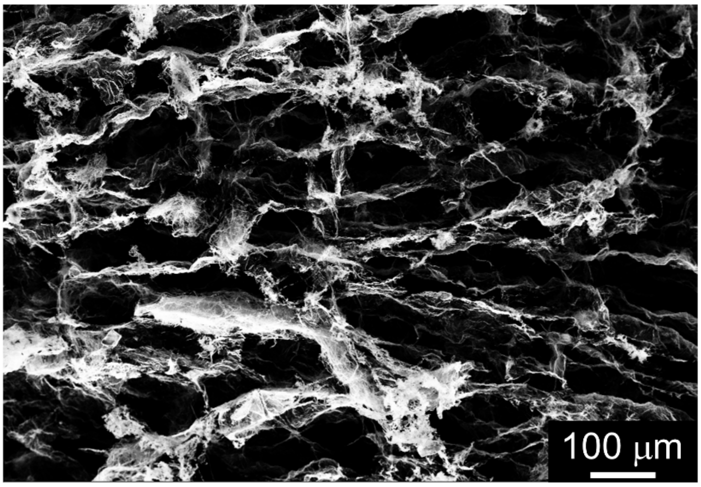

Supplementary Figure 29. SEM images of GmA after 5 min open-flame test.

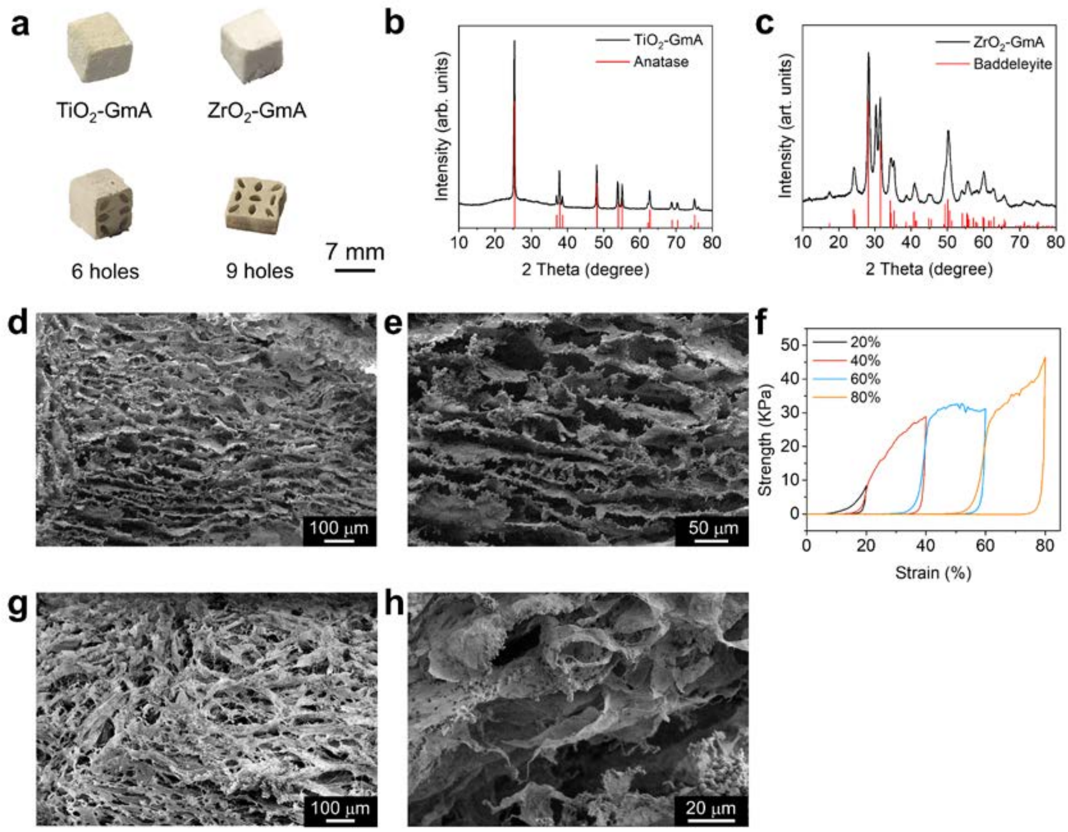

Supplementary Figure 30. Ceramic aerogels. **a** Photos of ceramic aerogels of TiO_2 and ZrO_2. **b, c** XRD patterns of ceramic aerogels. **b** TiO_2-GmA and anatase, **c** ZrO_2-GmA and baddeleyite. **d, e** SEM images of TiO_2-GmA. **f** Compressive stress-strain curves of the TiO_2-GmA. **g, h** SEM images of ZrO_2-GmA.

With the GmAs as templates, ceramic aerogels (e.g., TiO_2 and ZrO_2) can be prepared by the means of chemical deposition and calcination process (Detail procedures please refer to method section). The as-prepared ceramic aerogels well match the XRD patterns anatase TiO_2 and baddeleyite ZrO_2 (JCPDS 21-1272 and JCPDS 37-1484), demonstrating the successful fabrication of ceramic aerogels assisted by the ultralight templates. Meanwhile, the microstructures of the ceramic aerogels well duplicate the sheet-like structures of graphene layer (Supplementary Fig. 30). But to be noted, the strength of ceramic aerogels still needs to be improved (Supplementary Fig. 30f), which is excluded in this work because it is a systematic work involving the optimization of the complex calcination and crystallization process.

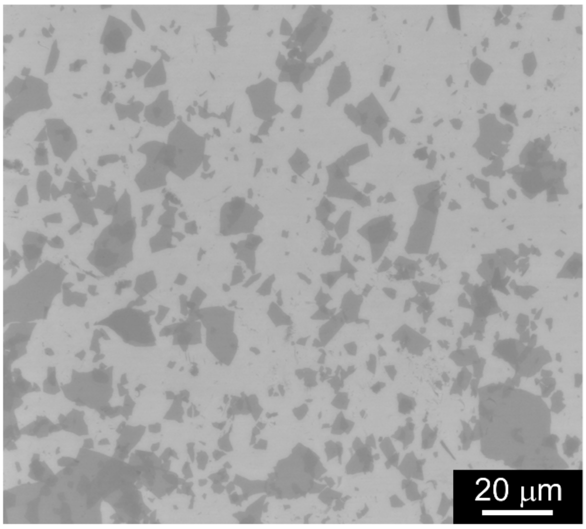

Supplementary Figure 31. SEM images of GO sheets with an average lateral size of 8-12 μm.

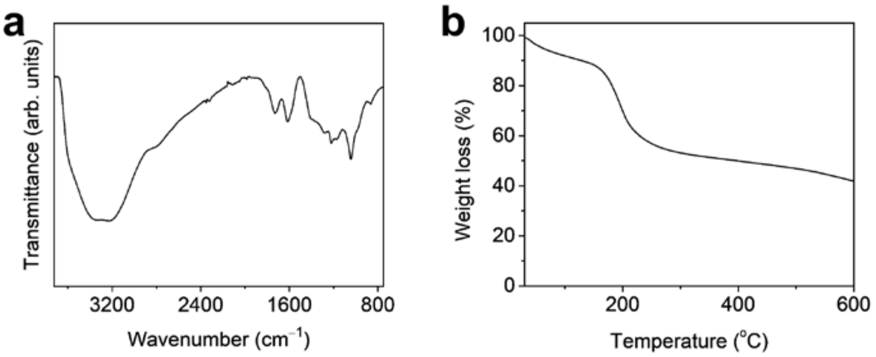

Supplementary Figure 32. Chemical characterizations of GO. **a** ATR-FTIR spectrum of GO. **b** TGA curve of GO.

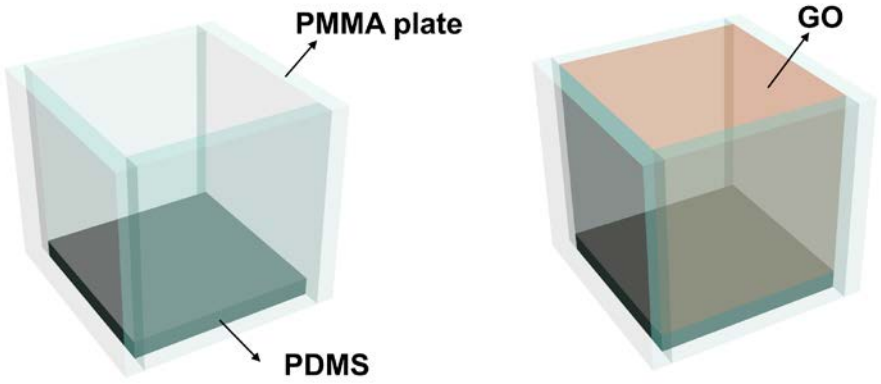

Supplementary Figure 33. Frozen mold consisting of polymethyl methacrylate (PMMA) plates and PDMS layer.

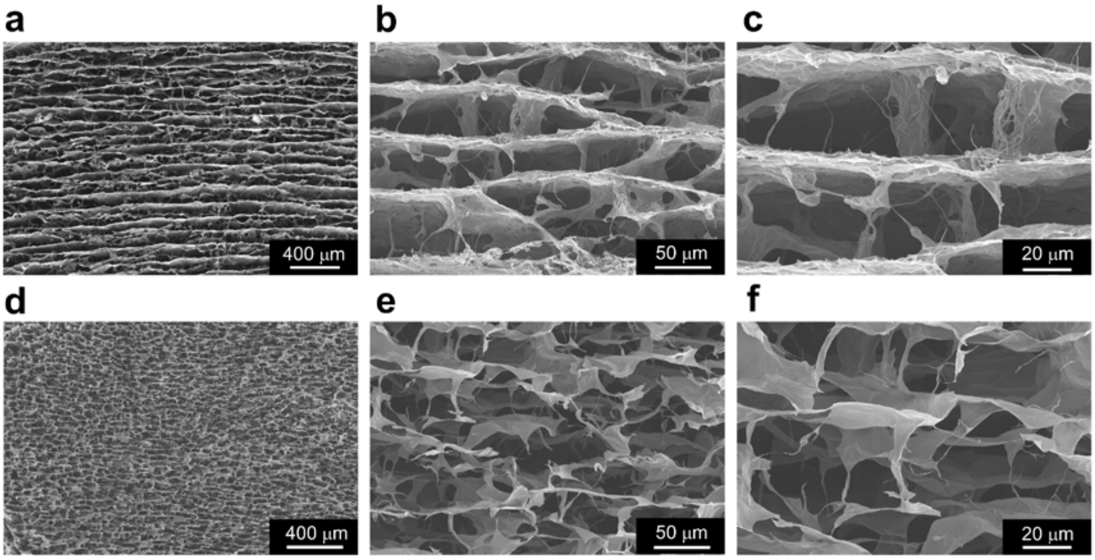

Supplementary Figure 34. Cross-section SEM images of GmA and PGA. **a, b, c** GmA at different scales, and **d, e, f** PGA at different scales.

Both GmA and PGA were fabricated by the same procedures including directional freeze-drying and thermal annealing process. Accordingly, the graphene walls of GmA and PGA both show orientation arrangement due to the squeezing of the ice crystals. While, the degree of the orientation mainly relies on the gelation behavior of the initial dispersion. The pure GO sheets with strong interaction would restrict the ice growth, thus leading to a more random distribution after freeze-drying. And combining with PI nanofibers, the van de waals forces between the GO sheets would be partially shielded, which contributes to align the GO/PI composites along with water-ice interface during the directional growth process of ice. Thus, the GmA exhibits a more ordered and thick graphene walls than that of PGA.

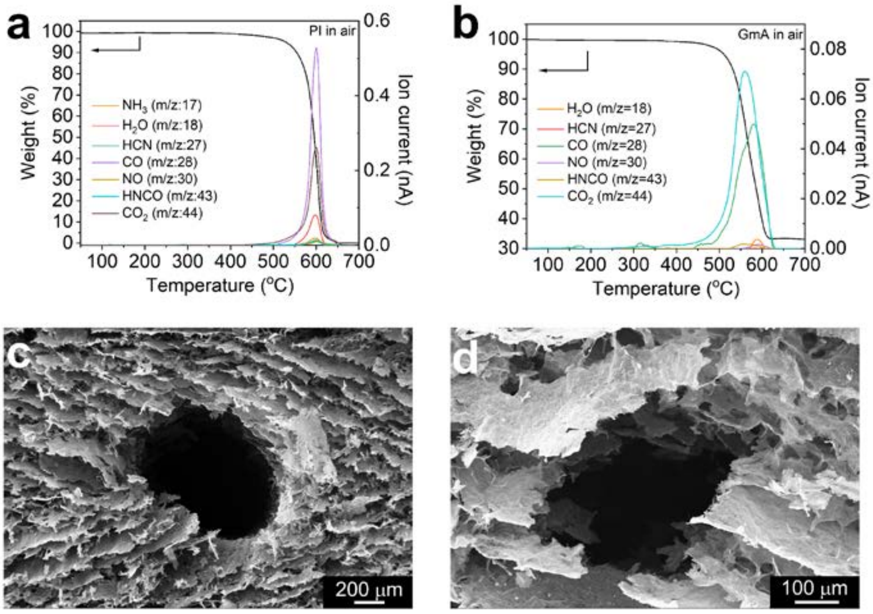

Supplementary Figure 35. TGA-MS spectrum. **a** PI in air. **b** GmA in air. SEM images of GmA surfaces after laser-cutting and tearing. **c, e, g** GmA surfaces after laser-cutting under different magnification. **d, f, h** GmA surfaces after tearing under different magnification.

In general, laser processing involves rapid energy input and energy deposition on a solid's surface. The pulsed/continuous laser energy is absorbed by the electrons in this process. Then the electrons interact with the lattice to complete the energy transfer (10^{-11}–10^{-12} s). And the thermal equilibrium is established between the lattices with increasing temperature and kinetic energy of the lattice. The resultant temperature depends on the absorbed energy, thermal diffusion rate of materials, and work conditions during laser irradiation. In our experiment, the average temperature of the laser spot is about 1000 °C, and the instantaneous temperature of laser is as high as 1300 °C, which is measure by a thermocouple with a metal probe. At this temperature, the graphene and PI will be rapidly oxidized by oxygen. The thermogravimetric analysis/mass spectrometry (TGA-MS) measurement confirmed that once the temperature exceeds 500 °C, the PI and GmA would generate a lots of combustion products, such as CO_2 (m/z:44), CO (m/z:28), H_2O (m/z:18), and a small amount of nitride (Supplementary Fig. 35a, b). During this process, the GmA is sculpted with hole structures (Supplementary Fig. 35c). However, the fast energy input of laser often makes the spot enlargement and have a wider engraving area. The smallest laser-engraving size that this nanosecond laser machine can achieve is 200 μm. And, the precise processing resolution is about 1 mm.

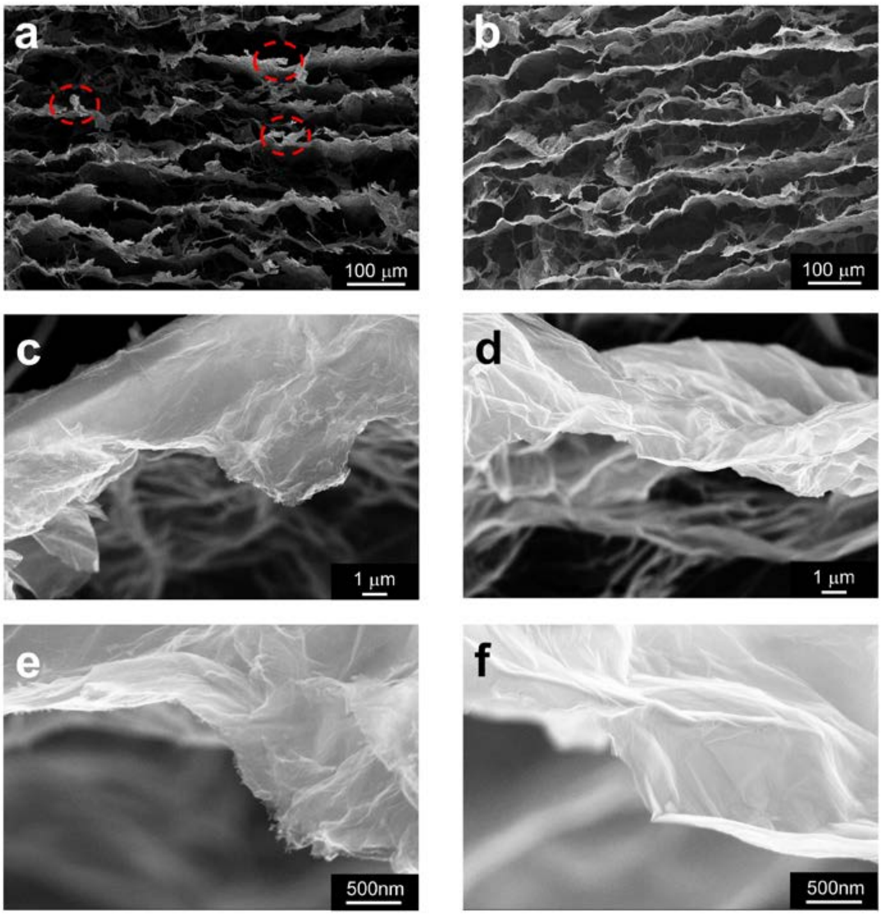

Supplementary Figure 36. SEM images of GmA surfaces after laser-cutting and tearing. **a, c, e** GmA surfaces after laser-cutting under different magnification. **b, d, f** GmA surfaces after tearing under different magnification.

At the surface of graphene layers, the gases generated from the rapid combustion of graphene and PI will produce fast-flowing airflow that can break the graphene walls and result in partially cracked structures (Marked in red circles in Supplementary Fig. R36a). In contrast, the GmA surface ripped by hands exhibits a relatively complete structure without obvious fractures (Supplementary Fig. 36b). Additionally, because the temperature at the edge of the laser may be lower than that at the spot center. Some insufficient combusted flocs are often left on the laser-engraving surface (Supplementary Fig. 36c, e). In contrast, the tearing sufaces of graphene walls show a smooth structure (Supplementary Fig. 36d, f).

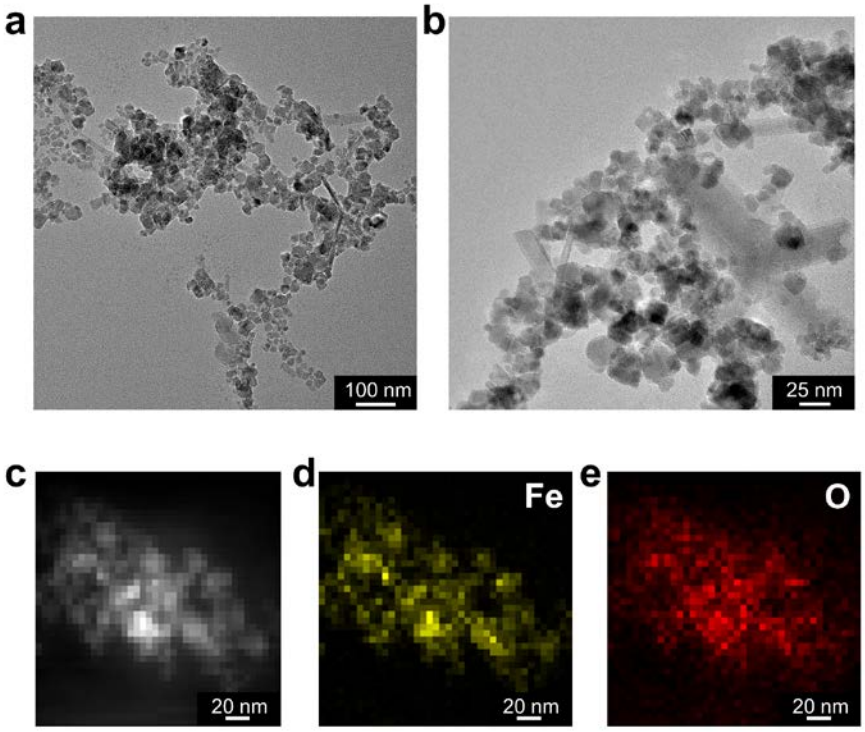

Supplementary Figure 37. Structure characterizations of Fe_3O_4 nanoparticles. **a, b** TEM images of Fe_3O_4 nanoparticles. **c-e** Energy dispersive spectroscopy mapping of corresponding area.

S3. Supplementary Table

Supplementary Table 1. Comparisons of the compressive performances of carbon-based aerogels.

Materials	Density (mg cm^{-3})	Strain (%)	Cycles	Strain retention (%)	Stress retention (%)	Ref.
GmA	0.10-6.52	30	5000	100	98	This work
		50	1000	100	95.3	This word
		80	1000	98	82	This word
GmA$_{100}$[a]		50	900	100	99	This word
GmA$_{100}$		80	900	99	90	This word
	0.10	Elasticity 80% strain for 50 cycles				This word
BCNF$_2$@RF[b]	9.2	30	10000	98.5	98	3
		50	10000	98	93	3
		70	1000	92	74	3
UFAs[c]	1	50	1000	100	88	4
	0.16	inelasticity				4
IGA[d]	12	50	1000	98	90	5
BC-CNFAs[e]	3.0-18.3	40	1000000	95.5	91	6
		60	500000	92	96	6
3DPGA[f]	0.5-10	50	10	98	77	7
BGFs[g]	3.8	50	10	97	65	8
C-CNC/rGO[h]	1.64	50	1000	85	62	9
		50	10000	91.8	57.7	9
BGAs[i]	5-16.2	50	100	100	92	10
AGAs[j]	1.29	50	10	97	94.8	11
OGF[k]	13.2	60	500	82	71.2	12
GCMs[l]	5.1	80	1000	93	76	13
BDF rG-O[m]	8	50	1000	92	87.5	14
ULGA[n]	3	50	1000	100	70	15
C-G monliths[o]	14.1	50	250000	98	86	16
		80	10000	93	60	16
GSs[p]	5.0-7.8	70	1000	90	70	17
KGM-CNFAs[q]	5.2	50	1000	95.7	75	18
EGC-PPY[r]	/	80	100	80	90	19
GEs[s]	0.16	Elasticity 50% strain				20

[a]GmA_{100} = GmA after precompression of 100 cycles.
[b]$BCNF_2$@RF = bacterial cellulose nanofiber with resorcinol-formaldehyde as hard carbon
[c]UFAs = ultra-flyweight aerogels
[d]IGA = integrated graphene aerogels
[e]BC-CNFAs = carbon nanofiber aerogels derived from bacterial cellulose (BC)
[f]3DPGA = three dimensional printed graphene aerogel
[g]BGFs = bubble-derived graphene foams
[h]C-CNC/rGO = carbon aerogels with cellulose and reduced graphene oxide
[i]BGAs = biomimetic graphene aerogels
[j]AGAs = anisotropic graphene aerogels
[k]OGF = ordered microstructure graphene foam
[l]GCMs = graphene-based cellular monoliths
[m]BDF rG-O = bidirectional freezing rGO
nULGA = ultralight graphene aerogel
[o]C-G monliths = graphene cellular monoliths
[p]GSs = graphene aerogel spheres
[q]KGM-CNFAs = carbonaceous nanofibrous aerogels derived from konjac glucomannan (KGM)
[r]EGC-PPY = elastin-gelatin-carbon nanotube polypyrrole
[s]GEs = graphene elastomers

Supplementary Table 2. GmA with transverse deformation (ε_{22}) variation under different longitude applied strain (ε_{11}).

ε_{11}	ε_{22} $GmA_{-1.2}$	ε_{22} GmA_{-1}	ε_{22} $GmA_{-0.7}$	ε_{22} $GmA_{-0.4}$	ε_{22} GmA	ε_{22} GmA_{+6}	ε_{22} GmA_{+5}	ε_{22} GmA_{+4}	ε_{22} GmA_{+3}
0%	0%	0%	0%	0%	0%	0%	0%	0%	0%
10%	−4.7%	−8.1%	−2.6%	−0.1%	−0.3%	16.4%	14.5%	11.6%	8.5%
20%	−19.0%	−18.8%	−6.1%	−0.2%	0%	23.3%	24.1%	17.4%	10.5%
30%	−26.7%	−23.5%	−10.4%	−0.6%	−0.2%	28.8%	28.6%	21.3%	14.3%
40%	−28.4%	−22.1%	−11.7%	−0.5%	−0.3%	30.1%	31.5%	24.0%	14.3%
50%	−25.4%	−17.6%	−7.8%	−0.3%	−0.6%	35.6%	32.4%	23.6%	14.3%
60%	−19.4%	−14.7%	−8.7%	0%	−0.5%	39.7%	32.4%	22.1%	14.0%
70%	−5.2%	−2.6%	−5.7%	0.4%	0.8%	43.8%	32.8%	23.3%	14.2%
80%	6.9%	5.5	2.6%	0.8%	1.2%	46.9%	34.0%	26.4%	13.2%

Supplementary Table 3. Comparisons of the Poisson's ratio of GmA with other carbon-based aerogels.

Materials	Poisson's ratio	Ref.
GmA	Tunable $-0.95 < v < 1.64$	This work
UFAs[a]	negative Poisson's ratio	4
IGA[b]	negative Poisson's ratio	5
GSs[c]	positive Poisson's ratio	17
KGM-CNFAs[d]	zero Poisson's ratio	18
NDGA[e]	Tunable $-0.3<v<0.46$	21
Aerographite	positive Poisson's ratio	22
RGO/GNP[f]	zero Poisson's ratio	23
Hyperbolic GM[g]	negative Poisson's ratio	24
3DGS[h]	zero Poisson's ratio	25

[a]UFAs = ultra-flyweight aerogels
[b]IGA = integrated graphene aerogels
[c]GSs = graphene aerogel spheres
[d]KGM-CNFAs = carbonaceous nanofibrous aerogels derived from konjac glucomannan (KGM)
[e]NDGA = natural drying graphene aerogel
[f]RGO/GNP = reduced graphene oxide and graphene nanoplatelets
[g]Hyperbolic GM = hyperbolically patterned graphene metamaterial
[h]3DGS = three dimensional graphene sponge

S4. Supplementary References

1. Plimpton, S. Fast parallel algorithms for short-range molecular dynamics. *J. comput. physics*, **117**, 1-19 (1995).
2. Cranford, S. & Buehler, M. J. Twisted and coiled ultralong multilayer graphene ribbons. *Model. Simul. Mater. Sc.* **19** 054003 (2011).
3. Yu, Z-L., et al. Superelastic hard carbon nanofiber aerogels. *Adv. Mater.* **31**, 1900651 (2019).
4. Sun, H., Xu, Z. & Gao, C. Multifunctional, ultra-flyweight, synergistically assembled carbon aerogels. *Adv. Mater.* **25**, 2554-2560 (2013).
5. Liu, J., Liu, Y., Zhang, H-B., Dai, Y., Liu, Z. & Yu, Z-Z. Superelastic and multifunctional graphene-based aerogels by interfacial reinforcement with graphitized carbon at high temperatures. *Carbon* **132**, 95-103 (2018).
6. Li, C., et al. Temperature-invariant superelastic and fatigue resistant carbon nanofiber aerogels. *Adv. Mater.* **32**, 1904331 (2020).
7. Zhang, Q., Zhang, F., Medarametla, S. P., Li, H., Zhou, C. & Lin, D. 3D printing of graphene aerogels. *Small* **12**, 1702-1708 (2016).
8. Zhang, R., et al. A bubble-derived strategy to prepare multiple graphene-based porous materials. *Adv. Funct. Mater.* **28**, 1705879 (2018).
9. Zhuo, H., et al. A supercompressible, elastic, and bendable carbon aerogel with ultrasensitive detection limits for compression strain, pressure, and bending angle. *Adv. Mater.* **30**, 1706705 (2018).
10. Yang, M., et al. Biomimetic architectured graphene aerogel with exceptional strength and resilience. *ACS Nano* **11**, 6817-6824 (2017).
11. Liu, T., Huang, M., Li, X., Wang, C., Gui, C-X. & Yu, Z-Z. Highly compressible anisotropic graphene aerogels fabricated by directional freezing for efficient absorption of organic liquids. *Carbon* **100**, 456-464 (2016).
12. Yao, B., Chen, J., Huang, L., Zhou, Q. & Shi, G. Base-induced liquid crystals of graphene oxide for preparing elastic graphene foams with long-range ordered microstructures. *Adv. Mater.* **28**, 1623-1629 (2016).
13. Qiu, L., Liu, J. Z., Chang, S. L., Wu, Y. & Li, D. Biomimetic superelastic graphene-based cellular monoliths. *Nat. Commun.* **3**, 1241 (2012).
14. Wang, C., et al. Freeze-casting produces a graphene oxide aerogel with a radial and centrosymmetric structure. *ACS Nano* **12**, 5816-5825 (2018).
15. Hu, H., Zhao, Z., Wan, W., Gogotsi, Y. & Qiu, J. Ultralight and highly

compressible graphene aerogels. *Adv. Mater.* **25**, 2219-2223 (2013).

16. Gao, H. L., et al. Super-elastic and fatigue resistant carbon material with lamellar multi-arch microstructure. *Nat. Commun.* **7**, 12920 (2016).

17. Zhao, X., Yao, W., Gao, W., Chen, H. & Gao, C. Wet-spun superelastic graphene aerogel millispheres with group effect. *Adv. Mater.* **29**, 1701482 (2017).

18. Si, Y., Wang, X., Yan, C., Yang, L., Yu, J. & Ding, B. Ultralight biomass-derived carbonaceous nanofibrous aerogels with superelasticity and high pressure-sensitivity. *Adv. Mater.* **28**, 9512-9518 (2016).

19. Liu, Y., et al. Highly flexible and resilient elastin hybrid cryogels with shape memory, injectability, conductivity, and magnetic responsive properties. *Adv. Mater.* **28**, 7758-7767 (2016).

20. Qiu, L., et al. Extremely low density and super-compressible graphene cellular materials. *Adv. Mater.* **29**, 1701553 (2017).

21. Xu, X., Zhang, Q., Yu, Y., Chen, W., Hu, H. & Li, H. Naturally dried graphene aerogels with superelasticity and tunable poisson's ratio. *Adv. Mater.* **28**, 9223-9230 (2016).

22. Mecklenburg, M. et al. Aerographite: ultra lightweight, flexible nanowall, carbon microtube material with outstanding mechanical performance. *Adv. Mater.* **24**, 3486-3490 (2012).

23. Yang, J. et al. Air-dried, high-density graphene hybrid aerogels for phase change composites with exceptional thermal conductivity and shape stability. *J. Mater. Chem. A* **4**, 18067-18074 (2016).

24. Zhang, Q., et al. Hyperbolically patterned 3D graphene metamaterial with negative Poisson's ratio and superelasticity. *Adv. Mater.* **28**, 2229-2237 (2016).

25. Zhao, K., et al. Super-elasticity of three-dimensionally cross-linked graphene materials all the way to deep cryogenic temperatures. *Sci. Adv.* **5**, eaav2589 (2019).
